# Usefulness and Ease of Use of an Artificial Intelligence Chatbot for Kidney Stone Formers

**DOI:** 10.7759/cureus.77398

**Published:** 2025-01-13

**Authors:** Mulun Huang, Kymora Scotland

**Affiliations:** 1 Department of Urology, University of California Los Angeles, Los Angeles, USA

**Keywords:** ai chatbot, health education, health technology, kidney stone, large language model

## Abstract

Background

Kidney stone formers need to navigate an overwhelming amount of information for acute stone management and stone recurrence prevention. Artificial intelligence (AI) chatbots may be uniquely equipped to support stone formers in this process. UroGPT™ (Dornier Medtech, Wessling, Germany) is an AI chatbot developed to answer kidney stone-related questions and help kidney stone formers manage their care. The aim of this study is to evaluate the perceived usefulness and ease of use of UroGPT™ among kidney stone formers.

Methods

A pre-survey and a post-survey were designed to assess participants’ experiences with their sources of kidney stone-related information before using UroGPT™, and their experiences using UroGPT™, respectively.

Results

In all, 626 and 260 participants participated in the pre- and post-survey, respectively. Among post-survey participants, 190 (73.08%) strongly agreed that using UroGPT™ makes it easier for them to get the information they need about kidney stones, 183 (70.38%) strongly agreed that they are satisfied with UroGPT™ for improving their understanding of kidney stones, and 218 (83.85%) strongly agreed that they find UroGPT™ easy to use. UroGPT™ achieved a total Net Promoter Score (NPS) of +75.77.

Conclusion

This study found that UroGPT™ is generally easy to use and useful for kidney stone formers. A high NPS indicates the potential for UroGPT™ to be widely adopted in kidney stone former communities.

## Introduction

Kidney stone formers concurrently face the challenges of navigating the acute management of kidney stones and preventing stone recurrence long-term. The amount of information kidney stone formers need to absorb in order to optimally take care of their health amid these challenges can be overwhelming. Not only do kidney stone formers need to sufficiently understand various surgical and medical management options for stone treatment, they also need to maintain motivation and develop self-management skills in engaging with their treatment and lifestyle modification best practices.

Physician consultations alone, due to their limited durations and relative infrequencies, often cannot completely address the needs of kidney stone formers for consistently accessible reliable information as well as support for navigating stone management and prevention on a regular basis [1]. Digital health tools, specifically artificial intelligence (AI) chatbots, are uniquely equipped to address these needs for kidney stone formers.

Already, many health consumers, including kidney stone formers, seek online health information to manage their health. Across disease categories, 75% of consumers turn to the internet first in their search for health information [2]. Kidney stone formers are increasingly using online platforms, especially in their searches for information on diet, general kidney stone knowledge, and pain management [3]. Current forms of online health information, however, have several shortcomings. A study that evaluated the reliability of popular online content for surgical management options of kidney stones found that the quality of this content is moderate at best and that it lacks information pertinent to helping patients with decision-making [4]. Furthermore, online information is usually static, and not tailored to a specific person and their needs. Lastly, there is often an overwhelming amount of information available, some of it contradictory, which can be confusing and stressful for kidney stone formers to navigate. 

AI chatbots have the potential to overcome these shortcomings. First, they can be trained on a collection of curated, reliable information on kidney stones and generate logically synthesized output from relevant information. Secondly, they can supply on-demand information targeted specifically to a kidney stone former’s question or request through direct interaction with the user. Research has shown that kidney stone formers are interested in using interactive health platforms. These platforms have been shown to improve health outcomes for kidney stone formers. Specifically, 76% of kidney stone patients in a survey were interested in using an app or device to better adhere to kidney stone prevention guidelines, and 88% were interested in using mobile health technology to increase their fluid consumption [5]. A small pilot study found that an online program where kidney stone formers received daily, immediate feedback on compliance with their diet recommendations significantly reduced urine oxalate levels in participants [6]. AI chatbots can not only answer knowledge-based questions, they can also complete tasks that make kidney stone knowledge more actionable for users, for example, creating a stone prevention diet plan based on a particular user’s preferences and access to resources. Based on their preferences, users can also adjust certain characteristics, such as the complexity and density, of the information AI chatbots provide by directly “chatting” with the chatbot. Lastly, AI chatbots have the potential to make users feel more empathized with while receiving kidney-stone-related information. In a study that compared physician-written and AI-generated replies to patients’ inbox messages, AI-generated replies were rated to be almost 10 times more empathetic than physician-written responses [7]. As AI chatbots become more prevalent in medicine, they have shown promising benefits in other disease areas. All patients (100%) in the PROSCA (prostate cancer communication assistant) trial who used a chatbot developed for prostate cancer awareness expressed willingness to use a medical chatbot in the future [8].

UroGPT™ (Dornier Medtech, Wessling, Germany) is an AI chatbot developed by an online education and engagement platform for kidney stone formers, tailored to answer kidney stone-related questions and help kidney stone formers manage their care. It was trained on kidney stone educational material validated by urologists. The purpose of this study is to evaluate the usefulness and ease of use of UroGPT™ among kidney stone formers.

This article was previously presented as a poster at the Abstracts of the 41st World Congress of Endourology and Urotechnology (WCET) 2024, August 12-16, 2024.

## Materials and methods

UroGPT development

UroGPT™ was developed by customizing the responses of GPT-4, a Large Language Model developed by Open AI, through Microsoft Azure Open AI Service, which provides access to Open AI through Microsoft Azure’s Health Insurance Portability and Accountability Act (HIPAA)-compliant platform [9]. The customization process involved (1) augmenting its knowledge base with an existing database of kidney stone-related, domain-specific knowledge verified by board-certified urologists, including patient-facing educational materials, for the model to generate accurate and contextually relevant information through the Retrieval Augmented Generation process in response to users’ questions and requests, and (2) prompting with instructions tailored to develop an empathetic personality in UroGPT™, allowing it to be adept at recognizing emotional cues from users and delivering support in a manner that mirrors the compassionate communication valued in healthcare.

UroGPT™ was published to be accessible for free on an online education and engagement platform for kidney stone formers [10].

Study design

Survey Design

A pre-survey and a post-survey were designed to anonymously assess participants’ experiences with their sources of kidney stone-related information before using UroGPT™, and their experiences using UroGPT™, respectively. The pre-survey was a two-question survey that asked participants to select their current sources of kidney stone-related information and then asked them to rate their level of satisfaction with those sources (Appendix A). The post-survey was a five-question survey (Appendix B). Four questions asked the participants to rate their level of agreement or disagreement with different statements on the usefulness and ease of use of UroGPT™. These questions were adapted from the perceived usefulness and perceived ease-of-use domains of the Health-IT Usability Evaluation Scale (Health ITUES), a validated instrument developed specifically to evaluate the usability of digital health tools [11]. The scale was designed for the wordings of predefined parts of certain questions to be adaptable based on the specific digital health tool it is applied towards. Each question within the scale has been validated against the domain it seeks to measure, therefore allowing the selection of individual questions for adoption. One question was a Net Promoter Score (NPS) question, which asked participants to rate the likelihood of them recommending UroGPT™ to other kidney stone formers. All questions in the pre-survey and post-survey were multiple-choice questions. The study was granted exempt status by the Institutional Review Board of the University of California Los Angeles (IRB #23-000249).

Participant Recruitment and Survey Implementation

During the beta launch of UroGPT™, kidney stone formers who are members of a large, online kidney stone former community were informed about the study through targeted communications across email, social media, and notices on an online patient education and engagement platform for kidney stone formers. Community members who intended to utilize UroGPT™ were invited to participate in the study. Upon registering for UroGPT™ access, participants were invited to voluntarily and anonymously fill out the pre-survey, use UroGPT™, and fill out the post-survey on the website.

Data Analysis

NPS was calculated with the following formula: NPS = [(% Promoters) - (% Detractors)]*100. Promoters were defined as participants who rated a 9 or 10 on the NPS scale. Detractors were defined as participants who rated 1 to 6 on the NPS scale. Participants' answers to other multiple-choice questions in the pre-survey and post-survey were analyzed using descriptive statistics.

## Results

During a one-month period after the launch of UroGPT™ in March 2024, 626 participants participated in the pre-survey and 260 (41.5%) participated in the post-survey.

Among participants of the pre-survey (Table 1), 472 (75.4%) reported their healthcare provider as a usual source of information for kidney stones, 380 (60.7%) reported online search engines, 168 (26.8%) reported family and friends with kidney stones, 121 (19.3%) reported online patient communities and platforms, 109 (17.4%) reported social media channels, and 43 (6.8%) reported books, brochures and printed materials as usual sources of information on kidney stone. Of the participants, 28 (4.5%) indicated they had not looked for information on kidney stone disease before. In terms of levels of satisfaction with their usual sources of kidney stone-related information, 15 (2.4%) participants were very unsatisfied, 34 (5.4%) participants were somewhat unsatisfied, 204 (32.6%) participants were neutral, 233 (37.2%) participants were somewhat satisfied, 140 (22.4%) participants were extremely satisfied.

**Table 1:** Results of the pre-survey.

Pre-survey questions	Responses (N = 626)
Where do you usually get your information about kidney stones? Select all that apply.	
My healthcare provider (doctor, nurses, etc.)	472 (75.4%)
Online search engines (e.g. Google)	380 (60.7%)
Online patient communities and platforms (e.g. Worst Pain Ever, WPE Wellness)	121 (19.3%)
Social media channels (e.g. YouTube, Instagram)	109 (17.4%)
Family or friends with kidney stones	168 (26.8%)
Books, brochures, or printed materials	43 (6.8%)
I haven’t looked for information on kidney stones before	28 (4.5%)
Overall, how would you rate your satisfaction with your usual sources of kidney stone-related information?	
Very unsatisfied	15 (2.4%)
Somewhat unsatisfied	34 (5.4%)
Neutral	204 (32.6%)
Somewhat satisfied	233 (37.2%)
Extremely satisfied	140 (22.4%)

Among participants of the post-survey, 190 (73.08%) strongly agreed that using UroGPT™ makes it easier for them to get the information they need about kidney stones, 183 (70.38%) strongly agreed that they are satisfied with UroGPT™ for improving their understanding of kidney stones, 215 (82.69%) strongly agreed that they think UroGPT™ would be helpful for people with kidney stones, and 218 (83.85%) strongly agreed that they find UroGPT™ easy to use. Additional survey results are shown in Table 2. UroGPT™ achieved a total NPS of +75.77 (Table 3).

**Table 2:** Results of the post-survey.

Post-survey questions	Responses (N=260)
Using UroGPT makes it easier to get the information I need about kidney stones.	
Strongly disagree	0
Somewhat disagree	3 (1.15%)
Neither agree or disagree	16 (6.15%)
Somewhat agree	51 (19.62%)
Strongly agree	190 (73.08%)
I am satisfied with UroGPT for improving my understanding of kidney stones.	
Strongly disagree	0
Somewhat disagree	3 (1.15%)
Neither agree or disagree	17 (6.54%)
Somewhat agree	57 (21.92%)
Strongly agree	183 (70.38%)
I think UroGPT would be helpful for people with kidney stones.	
Strongly disagree	0
Somewhat disagree	1 (0.38%)
Neither agree or disagree	7 (2.69%)
Somewhat agree	37 (14.23%)
Strongly agree	215 (82.69%)
I find UroGPT easy to use.	
Strongly disagree	1 (0.38%)
Somewhat disagree	2 (0.77%)
Neither agree or disagree	11 (4.23%)
Somewhat agree	28 (10.77%)
Strongly Agree	218 (83.85%)
On a scale of 1 to 10, how likely are you to recommend UroGPT to another kidney stone former?	
1	1 (0.38%)
2	1 (0.38%)
3	1 (0.38%)
5	6 (2.31%)
6	3 (1.15%)
7	11 (4.23%)
8	28 (10.77%)
9	42 (16.15%)
10	167 (64.23%)

**Table 3:** NPS summative results. NPS: Net Promoter Score.

NPS summative results	
Average rating (out of 10)	9.24
Promoters (% rated 9 or 10)	80.38%
Detractors (% rated 1 to 6)	4.62%
NPS score	75.77%

## Discussion

To our knowledge, UroGPT™ is the first chatbot designed specifically for providing kidney stone formers with kidney-stone-related information. Survey results showed that UroGPT™ is generally easy to use and useful for kidney stone formers, especially in helping them find and understand information on kidney stones. A high NPS indicated the potential for UroGPT™ to be widely adopted in kidney stone formers communities. This result is consistent with studies on consumer-facing AI chatbots for other disease areas in urology, such as the PROSCA trial [8,12]. New data from the PROSCA trial found that 73% of participants agreed that a chatbot aimed to provide users with information on prostate cancer improved their informedness about prostate cancer and 91% of participants would use the chatbot again [12]. Recent studies in other disease areas have also found similarly positive user feedback for the usability of chatbots in providing personalized health information, such as in weight loss and radiation therapy [13,14]. Our findings add to this existing literature and further suggest the promise for AI chatbots to be adopted broadly in providing health information to consumers. 

Interestingly, the PROSCA trial additionally found that users of the chatbot had a significantly decreased need for information overtime as compared to the control group [12]. This finding points to a potential for the information-provision capabilities of medical AI chatbots, including UroGPT™, to play a role in patient-provider communications. In our study, prior to the use of UroGPT™, 75.4% of participants indicated their healthcare provider as a source where they usually get information about kidney stones. However, only 59.6% of participants indicated that they were somewhat or extremely satisfied with their current sources of kidney stone-related information. It would be interesting for future research to investigate whether UroGPT™ can play a role in relieving provider workload and improving patients’ overall satisfaction with the information they receive about kidney stones. 

Finally, because of the versatility of AI chatbots, UroGPT™ has the potential to provide kidney stone formers with more than kidney-stone-related information. A study on a chatbot that checks in with patients with gynecologic malignancies during chemotherapy found that it reduced rates of emergency department visits and unscheduled hospitalizations [15]. A study on a chatbot designed to support patients with gastrointestinal cancers by not only providing information to users as prompted but also surveying their symptom changes was found to result in high engagement from patients [16]. Developing additional features centered around user engagement could make UroGPT™ an even more powerful tool for kidney stone formers.

The main limitation of this study lies in that at the time of recruitment, participants knew that the study involved using UroGPT™ and answering questions regarding their user experience. Therefore, there was likely a selection bias towards kidney stone formers who have pre-existing interests in using AI chatbots and who have higher digital literacy. These participants are likely to find UroGPT™ easy to use and useful and provide a high NPS. While the results of this study are highly promising, future studies should further assess the usability and helpfulness of UroGPT™ among patients with diverse demographics and varying levels of digital literacy.

## Conclusions

UroGPT™, an AI chatbot developed to answer kidney stone-related questions and help kidney stone formers manage their care, is useful and easy to use for kidney stone formers, especially in helping them find and understand information on kidney stones. With 73.08% of post-survey participants strongly agreeing that UroGPT™ made it easier to obtain necessary information and 70.38% expressing satisfaction with the improvement in their understanding of kidney stones, UroGPT™ received an NPS of +75.77. These findings suggest significant potential for widespread adoption of UroGPT™ among kidney stone former communities. Future research should involve more diverse demographics and varying levels of digital literacy to further assess the usability and helpfulness of AI chatbots in kidney stone health education and navigation.

## Appendices

Appendix A: pre-survey questions

The table below outlines the pre-survey questions (Table 4).

**Table 4:** Pre-survey questions.

Pre-survey questions
1. Where do you usually get your information about kidney stones? Select all that apply.
My healthcare provider (doctor, nurses, etc.)
Online search engines (e.g. Google)
Online patient communities and platforms (e.g. Worst Pain Ever, WPE Wellness)
Social media channels (e.g. YouTube, Instagram)
Family or friends with kidney stones
Books, brochures, or printed materials
I haven’t looked for information on kidney stones before
2. Overall, how would you rate your satisfaction with your usual sources of kidney stone-related information?
Very unsatisfied
Somewhat unsatisfied
Neutral
Somewhat satisfied
Extremely satisfied

Appendix B: post-survey questions

The table below outlines the post-survey questions (Table 5).

**Table 5:** Post-survey questions.

Post-survey questions
1. Using UroGPT makes it easier to get the information I need about kidney stones.
Strongly disagree
Somewhat disagree
Neither agree or disagree
Somewhat agree
Strongly agree
2. I am satisfied with UroGPT for improving my understanding of kidney stones.
Strongly disagree
Somewhat disagree
Neither agree or disagree
Somewhat agree
Strongly agree
3. I think UroGPT would be helpful for people with kidney stones.
Strongly disagree
Somewhat disagree
Neither agree or disagree
Somewhat agree
Strongly agree
4. I find UroGPT easy to use.
Strongly disagree
Somewhat disagree
Neither agree or disagree
Somewhat agree
Strongly agree
5. On a scale of 1 to 10, how likely are you to recommend UroGPT to another kidney stone former?
1
2
3
5
6
7
8
9
10

## References

[1] Fritsche HM, Dötzer K (2012). Improving the compliance of the recurrent stone-former. Arab J Urol.

[2] Finney Rutten LJ, Blake KD, Greenberg-Worisek AJ, Allen SV, Moser RP, Hesse BW (2019). Online health information seeking among US adults: measuring progress toward a Healthy People 2020 objective. Public Health Rep.

[3] Kunitsky K, Takele R, Diaz P, Lim J, Patel PM, Scotland KB (2023). The evolution of kidney stone information available to patients: Interest trends of social media. Soc Int Urol J.

[4] Diaz P, Takele RA, Thaker S (2022). Kidney stone surgery: assessing public interest and evaluating social media content. J Endourol.

[5] Streeper NM, Lehman K, Conroy DE (2018). Acceptability of mobile health technology for promoting fluid consumption in patients with nephrolithiasis. Urology.

[6] Lange JN, Easter L, Amoroso R (2013). Internet program for facilitating dietary modifications limiting kidney stone risk. Can J Urol.

[7] Ayers JW, Poliak A, Dredze M (2023). Comparing physician and artificial intelligence chatbot responses to patient questions posted to a public social media forum. JAMA Intern Med.

[8] Görtz M, Baumgärtner K, Schmid T (2023). An artificial intelligence-based chatbot for prostate cancer education: design and patient evaluation study. Digit Health.

[9] Achiam J, Adler S, Agarwal S (2023). GPT-4 technical report. ArXiv.

[10] (2024). UroGPT™: the world’s first advanced AI chatbot for kidney stone support. https://wpewellness.worstpainever.com/urogpt.

[11] Yen PY, Wantland D, Bakken S (2010). Development of a customizable health IT usability evaluation scale. AMIA Annu Symp Proc.

[12] Baumgärtner K, Byczkowski M, Schmid T (2024). Effectiveness of the medical chatbot PROSCA to inform patients about prostate cancer: results of a randomized controlled trial. Eur Urol Open Sci.

[13] Rahmanti AR, Yang HC, Bintoro BS, Nursetyo AA, Muhtar MS, Syed-Abdul S, Li YJ (2022). SlimMe, a chatbot with artificial empathy for personal weight management: system design and finding. Front Nutr.

[14] Chow JC, Wong V, Sanders L, Li K (2023). Developing an AI-assisted educational chatbot for radiotherapy using the IBM Watson Assistant platform. Healthcare (Basel).

[15] Huang MY, Weng CS, Kuo HL, Su YC (2023). Using a chatbot to reduce emergency department visits and unscheduled hospitalizations among patients with gynecologic malignancies during chemotherapy: a retrospective cohort study. Heliyon.

[16] Gomaa S, Posey J, Bashir B (2023). Feasibility of a text messaging-integrated and chatbot-interfaced self-management program for symptom control in patients with gastrointestinal cancer undergoing chemotherapy: pilot mixed methods study. JMIR Form Res.

